# Determinants of cigarette smoking among adolescents in Ethiopia: A cross-sectional study

**DOI:** 10.18332/tid/110800

**Published:** 2019-09-02

**Authors:** Bereket Duko, Yirdaw Melese, Jemal Ebrahim

**Affiliations:** 1 Faculty of Health Sciences, College of Medicine and Health Sciences, Hawassa University, Hawassa, Ethiopia; 2 Department of Nursing, Rift Valley University, Hawassa, Ethiopia

**Keywords:** prevalence, determinants, risk factors, cigarette smoking, adolescents

## Abstract

**INTRODUCTION:**

Cigarette smoking is an important health hazard and major preventable cause of morbidity and mortality. This study aimed to assess the prevalence of cigarette smoking among Tabor secondary and preparatory school students in Hawassa City, Ethiopia, 2018.

**METHODS:**

A school-based cross-sectional study was conducted among 564 students aged 15–22 years using simple random sampling techniques, in the period 5–19 March 2018. Global Youth Tobacco Survey (GYTS) was used to assess smoking behaviours. Logistic regression analyses were employed to identify factors associated with cigarette smoking.

**RESULTS:**

The student prevalence of cigarette smoking was found to be 11% (95% CI: 8.5–13.9) of which 9.4% were current smokers. The proportion of cigarette smoking among male and female students was 8.2% and 2.8%, respectively. An age ≥18 years (AOR = 3.0, 95% CI: 1.29–7.00), students having friends who smoke (AOR= 4.04, 95% CI: 2.04–7.45), khat chewing (AOR=5.57, 95% CI: 2.44–12.76), alcohol consumption (AOR=4.14, 95% CI: 1.84–9.70) and illegal or illicit drug use (AOR=5.84, 95% CI: 1.96–17.36) were found to be significantly associated with cigarette smoking.

**CONCLUSIONS:**

Cost-effective programs that involve the participation of families, teachers and other stakeholders to deliver health education and which restrict accessibility, advertising and use of substances like alcohol, cigarettes, and other illicit drugs, are highly recommended.

## INTRODUCTION

Cigarette smoking refers to the practice of inhalation of the gases and hydrocarbon vapours generated by slowly burning tobacco in cigarettes^1^. Adolescence, from childhood to adulthood, is a complex maturing period involving natural and physical development and social interactions that may have short- and long-term consequences^2^.

Smoking causes the death of about 5 million individuals every year and is responsible for a very large share of the global burden of disease^3^. Studies in developed countries show that cigarette smoking has dramatically decreased in recent years^4^. Nevertheless, it is alarmingly increasing in low-income countries^5-8^. Nearly 80% of more than 1 billion smokers worldwide live in low- and middle-income countries, where the burden of tobacco-related illness and death is significant^9^. Tobacco use, is described as a ‘gate way’ to psychoactive substance and other illicit drug use among teenagers^10^. Findings from Global Youth Tobacco Surveillance revealed that the national prevalence of cigarette smoking among men living in sub-Saharan Africa ranges from 20% to 60% and tobacco use rates are increasing for both men and women annually^11^. According to the Ethiopian Demographic and Health Survey (EDHS) report of 2016, the prevalence of smoking in men and women is 4% and less than 1%, respectively^12^.

Exposure to smokers (friends, parents, teachers), availability of tobacco, low socioeconomic status, poor academic performance, low self-esteem, lack of perceived risk of use, and lack of skills to resist influences to tobacco use are factors that are associated with cigarette smoking among the youth^13-15^. Additionally, getting involved in physical fights, alcohol use, marijuana use and having sexual intercourse are also associated with cigarette smoking^7^.

Tobacco smoke contains in excess of 7000 synthetic substances and compounds. Hundreds are dangerous and in excess of 70 cause diseases like cancer. Adolescent bodies are more sensitive to nicotine, and adolescents are more easily addicted than adults. Antagonistic health impacts from regular cigarette smoking include an increased chronic cough, sputum production, wheezing and irritability, decreased concentration, increased appetite, and strong cravings for tobacco are common withdrawal symptoms^16^.

In recent years, Ethiopia has signed the WHO Framework Convention on Tobacco Control that bans the use of any tobacco products including cigarettes in any part of all indoor and outdoor areas that may have an impact on tobacco use among the youth^17^. This study was conducted to assess the prevalence of cigarette smoking and associated factors among adolescent students in Ethiopia, 2018.

## METHODS

### Study setting and population

This was a school-based cross-sectional study conducted in the period 5–19 March 2018 among Tabor secondary and preparatory school students in Hawassa City, Ethiopia. Students aged 15–22 years and in the regular program of education were included in the study. Among 6768 students, 600 were selected from grades 9 to 12 in the two schools. Thirty-six students were unable to give consent to participate in the study. The sample size was calculated using single population proportion formula with the assumptions: the proportion of cigarette smoking as 0.5, 95 % CI and margin error of 0.05, design effect of 1.5, and non-response rate of 0.05. Schools were stratified as secondary and preparatory schools and proportional allocation of students to these schools was applied. Cluster sampling technique was used to sample individual students and a specific number of students was proportionally allocated for each grade. Finally, by using cluster sampling technique, classrooms were randomly selected from each grade and each student was selected through simple random sampling techniques.

Ethical clearance was obtained from Hawassa University, College of Medicine & Health Sciences Institutional Review Board; Official permissions were also granted from the participating Tabor secondary and preparatory schools. Finally, written informed consent was obtained from each student after a clear explanation of the purpose of the study. Confidentiality of the information was ensured by the use of identification code variables in the questionnaire.

### Data collection

We used a self-administered questionnaire to gather the data. The survey questionnaire incorporated sociodemographic, economic and behavioral factors, and cigarette smoking habits. Global Youth Tobacco Survey (GYTS) questions were adjusted to the Ethiopian setting and used to assess smoking related habits^18^. GYTS comprises a core component and an optional component. The core component was used in all countries leading the Global Youth Tobacco Survey that takes into account worldwide investigation of results, and optional questions were used to address explicit issues in Ethiopia. The instrument was adapted to the Ethiopian context and was highly reliable in our pre-test study (Cronbach’s α=0.90)^18^.

### Data processing and analysis

The collected data were entered using statistical software Epi Info Version 3.5.1 and analyzed using SPSS version 22 statistical software. Student sociodemographic, economic and behavioral characteristics, and cigarette smoking habits, are described using statistics of frequency and percentage distributions. Measures of associations (odds ratio) are also applied to determine the prevalence of cigarette smoking and factors associated with it. Additionally, bivariate and multivariate analyses were conducted to determine odds ratios and confidence intervals.

## RESULTS

### Sociodemographic and substance-use characteristics of students

A total of 564 students participated in the study resulting in a response rate of 94%. Among students, 56.7% (320) were males, 53.4% (301) were aged >18 years, 29.8% (168) were from grade 9, 63.8% (360) had weekly pocket money of ≤3.5 US$, and 74.8% (422) were living with their parents. From the total students, 12.8% (72) ever used khat (Catha edulis), 21.6% (122) and 15% (85) had a history of ever alcohol use and alcohol use within the previous month of data collection, respectively (Table 1).

**Table 1:** Sociodemographic and substance use characteristics of students

*Variable*	*Frequency (n=564)*	*%*
**Sex**		
Male	320	56.7
Female	244	43.3
**Age group**		
<18	263	46.6
≥18 years	301	53.4
**Grade**		
9	168	29.8
10	139	24.6
11	153	27.1
12	104	18.4
**Weekly income**		
≤3.5	360	63.8
>3.5 US$	204	36.2
**Living with**		
Both parents	422	74.8
One parent	74	13.1
Relatives	35	6.2
Friends	29	5.1
Alone	4	0.7
**Current substance use**		
Khat	39	6.9
Alcohol	85	15.1
Illegal drugs	15	2.7
None	425	75.3
**Ever substance use**		
Khat	72	12.8
Alcohol	122	21.6
Illegal drugs	33	5.8
None	337	59.8

### Prevalence of cigarette smoking

**Figure 1:**
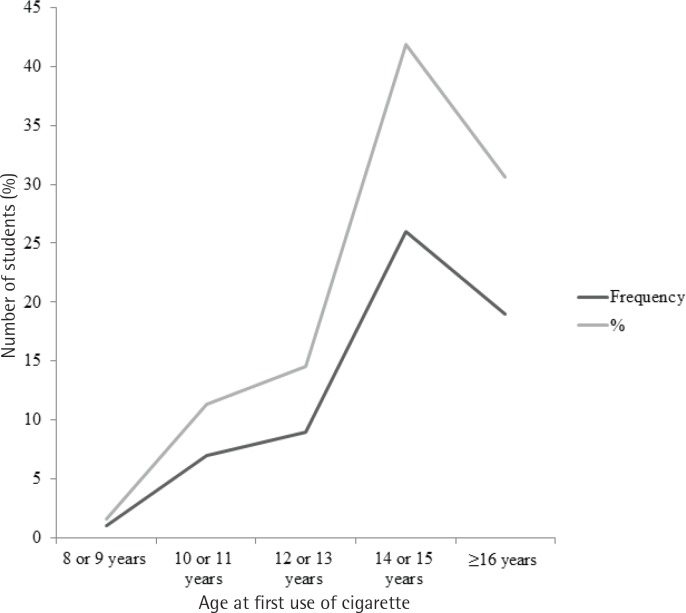
Age at first use of cigarettes in Tabor Secondary and Preparatory Schools students, Hawassa, Ethiopia, 2018

The overall prevalence of cigarette smoking in the study area was 11% (95% CI: 8.5–13.9). The proportion of cigarette smoking among male and female students was 8.2 % and 2.8%, respectively. Among cigarette smokers, 40.3% (25) and 29.7% (19) initiated smoking at age 14–15 years and ≥16 years, respectively (Figure 1).

### Exposure to others’ cigarette smoke

Twenty-six students confirmed that someone smoked inside their home during the past 7 days prior to this survey. Among students exposed to other’s cigarette smoke, 19% were exposed in enclosed public places for one or two days, and 4.3% were exposed for three to four days. Regarding outdoor public place smoking, 18.6% of students confirmed the presence of smoking for one or two days and 7.3% students noticed smoking occurring on all days within the 7 days prior to the data collection period (Table 2).

**Table 2:** Perception and practice of cigarette smoking (N=564 )

*Variable*	*Frequency (n=564 )*	*%*
**Days in which someone smoked inside home during the past 7 days**		
0	500	88.7
1 or 2	26	4.6
3 to 4	24	4.3
5 to 6	4	0.7
7 days	10	1.8
**Days in which someone smoked inside any enclosed public place, other than student’s home in the past 7 days**		
0	402	71.3
1 or 2	107	19
3 to 4	24	4.3
5 to 6 days	31	5.5
**Days in which someone smoked at any outdoor public place in the past 7 days**		
0	368	65.2
1 or 2	105	18.6
3 to 4	38	6.7
5 to 6	12	2.1
7 days	41	7.3
**During the past 30 days, someone smoked inside the school building or outside on school property**		
Yes	353	62.6
No	211	37.4
**Thinking about the smoke from other people’s tobacco smoking is harmful to the student**		
Definitely not	163	28.9
Probably not	37	6.6
Probably yes	32	5.7
Definitely yes	332	58.9
**In favor of banning smoking inside enclosed public places**		
Yes	447	79.3
No	117	20.7
**In favor of banning at outdoor public places**		
Yes	448	79.4
No	116	20.6

### Factors associated with cigarette smoking status

Binary logistic regression analysis revealed that students aged >18 years, having a friend who smoked, chewed khat, alcohol ever use, and history of illegal drug use were factors associated with cigarette smoking (Table 3).

**Table 3:** Factors associated with cigarette smoking status in students, Hawassa, Ethiopia, 2018 (n=564 )

*Predictor variables*	*Cigarette smoking*		*COR ( 95% CI)*	*AOR ( 95% CI)*	*p*
	*Yes*	*No*			
**Age group**					
<18	18	245	1	1	
≥18 years	44	257	2.33 (1.31–4.14)	3.0 (1.29–7.00)	0.011
**Grade**					
9	23	145	1	1	
10	15	124	0.76 (0.38–1.53)	0.96 (0.39–2.40)	0.932
11	19	134	0.89 (0.47–1.72)	0.44 (0.17–1.12)	0.085
12	5	99	0.32 (0.12–0.87)	0.11 (0.03–0.42)	0.001
**Having a friend who smokes cigarettes**					
Yes	42	122	6.54 (3.67–10.87)	4.04 (2.04–7.45)	0.01
No	20	380	1	1	
**Living with people who smoke**					
Yes	38	249	1.61 (0.76–7.87)	0.62 (0.23–5.73)	0.212
No	24	253	1	1	
**Ever use of khat**					
Yes	37	35	19.7 (10.7–36.4)	5.57 (2.44–12.76)	<0.001
No	25	467	1	1	
**Ever use of alcohol**					
Yes	43	79	12.1 (6.7–21.9)	4.14 (1.84–9.70)	0.001
No	19	423	1	1	
**Ever use of illegal drugs**					
Yes	25	8	41.72 (17.60–98.93)	5.84 (1.96–17.36)	0.002
No	37	494	1	1	
**Other people’s tobacco smoking is harmful**					
Definitely not	15	148	1.1 (0.57–2.12)	0.52 (0.22–1.24)	0.141
Probably not	15	22	7.4 (3.46–15.86)	2.23 (0.71–7.01)	0.168
Probably yes	4	28	1.55 (0.51–4.74)	1.77 (0.48–6.49)	0.388
Definitely yes	28	304	1		

## DISCUSSION

The study was conducted to assess the prevalence of cigarette smoking and associated factors among 564 secondary and preparatory school students who were selected through simple random sampling techniques, of critical importance to other similar studies and policy makers. In this study, the prevalence of cigarette smoking was 11% (95% CI: 8.5–13.9), in line with studies in Eastern Harar, Ethiopia^19^, Southwest Nigeria^20^, and Colombia^21^, but lower than that found in Addis Ababa, Butajira, Woreta, and Jimma, Ethiopia^22-25^, USA^15^, Harare in Zimbabwe, Khartoum, Johor in Malaysia and in Dubai secondary schools^15,26-28^, and in China^29^. Differences in prevalence might be related to study design, data collection tool, sample size, socioeconomic, cultural and participant variations. This study revealed that male adolescents were more likely to use tobacco than female adolescents; this is consistent with many reviewed studies conducted in Addis Ababa, Eastern Ethiopia & Butajira^22-25^. This might be due to the cultural and traditional background of the region where male students are more mobile and responsible for outdoor activities while females are usually responsible for activities inside their home. In addition, females have more familial connections such as taking care of family and other related social issues that hinder them from engaging in tobacco smoking.

In this study, cigarette smoking behavior is found to be more associated with students aged ≥ 18 years. This is in line with a study conducted in Harar, Ethiopia^20^. This shows that as the age of the adolescent increases the odds of testing or smoking increase. When the adolescent’s age increases, the possibility of being involved in risky behaviors tends to increase as a result of physiological and psychosocial changes that trigger smoking.

The odds of cigarette smoking are 5.57 times higher among khat chewers compared to those who do not chew khat. This is also true for drinking alcohol. It is also noticed that students who have a history of illegal drug use are 5.84 times more likely to use cigarettes than those who do not. A study conducted in Zimbabwe and other research support this evidence^15,29^. This may be because cigarettes, khat and alcohol are commonly interrelated.

Lastly, students who had friends who smoked cigarettes had a four-fold increase in the odds of using cigarettes compared to their counterparts. This finding is supported by different cross-sectional and case-control studies that reported the relationship between tobacco use and peer pressure conducted in Addis Ababa^30^, in Gondar, and elsewhere^31-33^. Having smoking friends could potentially influence both the initiation of smoking and maintaining smoking for a lifetime. In addition, some adolescents might link smoking cigarettes with daily social relationships with their friends.

Unlike other studies, factors like sex, educational performance, marital status, religion, average weekly pocket money, educational status of the mother and father, and parents’ marital status were not statistically significant with cigarette smoking status.

### Limitations

First, this study’s cross-sectional design does not allow causal associations to be made, hence further longitudinal research is required. Second, the smoking status of the participants was collected via self-administered questionnaires, allowing bias reporting and the possibility of error in the smoking prevalence among the students. Lastly, students are more likely to deny their cigarette smoking behaviors due to social desirability bias resulting in an underestimate of the prevalence of cigarette smoking.

## CONCLUSIONS

Prevalence of ever and current smoking cigarettes are found to be low among students in Tabor secondary and preparatory schools compared with many other studies conducted elsewhere. Moreover, factors like age group, having friends who smoke, khat chewing, alcohol consumption, and illegal drug use are found to be significantly associated with cigarette smoking. Cost-effective programs that involve the participation of families, teachers and other stakeholders to deliver health education and restrict accessibility, advertising and use of substances like alcohol, cigarettes and illicit drugs are highly recommended.
